# Daily photoprotection: What does it really mean?

**DOI:** 10.1111/phpp.12707

**Published:** 2021-06-29

**Authors:** Jean Krutmann, Corinne Granger, Carles Trullàs, Thierry Passeron

**Affiliations:** ^1^ IUF – Leibniz Research Institute for Environmental Medicine Düsseldorf Germany; ^2^ Medical Faculty Heinrich‐Heine‐University Düsseldorf Germany; ^3^ Innovation and development ISDIN Barcelona Spain; ^4^ CHU Nice Department of Dermatology University Côte d’Azur Nice France; ^5^ INSERM U1065 C3M University Côte d’Azur Nice France

## CONFLICT OF INTEREST

TP and JK are consultants for ISDIN; CG and CT are employees of ISDIN.

## AUTHOR CONTRIBUTIONS

TP and JK contributed to the conception of the article and critical revision for important intellectual content; CG and CT coordinated the article writing and revised it critically for important intellectual content. All authors gave final approval of the version to be published and agree to be accountable for all aspects of the work.

## ETHICAL APPROVAL

Not applicable.

## FUNDING INFORMATION

ISDIN

To the Editor,

The importance given to the cumulative effects of incidental, non‐extreme solar irradiation has lagged behind that given to severe episodic sunburn. Cumulative exposure is a contributor to precancerous skin lesions and carcinomas and plays a decisive role in (facial) skin ageing. While the concept of *daily photoprotection* has drawn more attention in recent years,^1^, ^2^ it seems that the intended meaning of the term is rather inconsistent: by some, it is used to refer to the daily use of broad‐spectrum sunscreens with sun protection factor (SPF) ≥ 50, generally in geographical areas where the ultraviolet index (UVI) is extreme, while by others it is used in reference to cosmetic products (day creams or make‐up) that provide variable levels of SPF as a secondary feature. Indeed, some countries (eg Australia) make regulatory distinctions between products that have sun protection as their primary or secondary function. The intended meaning may be influenced by the author's background: population factors such as frequency of skin phenotypes and local skin cancer rates, and geographical factors such as altitude, latitude, UVI and urban versus rural environments. At present, there is no consensus on what *daily photoprotection* means. This absence of a harmonized definition is confusing for practitioners and consumers and makes clear recommendations difficult.

Here, we venture that the most appropriate definition falls somewhere in the middle of the above extremes: routine daily use of a product that has, as its primary function, sun protection, in everyday conditions of non‐extreme exposure, and in adult population groups that do not fall into a high‐risk group. Within this context, we refer to topical products, which usually form the mainstay of protection. We consider daily photoprotection to be synonymous with daily sunscreen use for uncovered parts of the body, in addition to appropriate clothing, head covering and sunglasses. We also acknowledge that there is scope for oral agents as adjuvants due to their anti‐inflammatory, immunomodulatory and antioxidative actions,^3^ but do not discuss them here, instead focusing on daily‐use sunscreens.

To our understanding, daily photoprotection products, which can and should be optimized in terms of additional actives and cosmetic properties, form a subcategory of sunscreen rather than of day cream or make‐up. It is probably unrealistic to expect reliable reapplication several times a day; thus, pleasantly textured, durable, photostable products are necessary. Such products should reinforce the skin barrier and be non‐irritant, and UV filters should be included at the lowest effective concentration to minimize environmental impact.

Daily application represents an opportunity for protection against other environmental components of the skin ageing exposome: in urban environments, this means pollution in the form of traffic‐related particulate matter, and gases such as nitrogen dioxide and ground‐level ozone.^4^ Effective antioxidants and scavengers of reactive oxygen species can protect against this, in addition to their role against secondary mechanisms of photoaging. But, as stated, the primary function is sun protection; therefore, the ratio of protection against different wavelengths should reflect the significant—and synergistic—effects of radiation beyond the UVB range.^5^, ^6^ Daily photoprotection products should therefore offer substantial protection against UVA, and some protection against high‐energy visible light (HEVL) since these wavelengths penetrate the skin more deeply and induce effects including pigmentation and matrix metalloproteinase expression.^4^ Data on infrared A (IRA) are limited and have been debated,^7^ but given its suggested role in photoaging, it seems wise to include IRA protection until conclusive data are available.^8^ While the use of SPF ≥ 50 sunscreens is undoubtedly justified in certain conditions, these can be associated with poor cosmetic properties, which becomes highly relevant for compliance with daily use. It therefore seems unlikely that SPF ≥ 50 would offer substantial benefit in everyday conditions of non‐extreme UVI; SPF30 is probably adequate, though lower SPF may be suitable in low UVB circumstances such as winter in high‐latitude countries.^9^, ^10^ Since UVA and HEVL are not fully blocked by the atmosphere and are therefore more constant throughout the day (and year) than UVB, they remain factors that cannot be mitigated by avoiding midday sun and warrant a strong protection component. A more balanced UVA:UVB protection rather than the 1:3 ratio required for broad‐spectrum labelling claims in Europe and other countries should be the target. For HEVL‐induced pigmentation, the superior protection provided by tinted sunscreens is something of a two‐edged sword, their inherent colour providing a degree of cosmetic coverage that is not desirable to all. Infrared A protection is currently limited to the use of antioxidants, and the development of suitable filter molecules would be welcome. We hope that regular use of such sunscreen products will become established as daily routine.

## Data Availability

Data sharing is not applicable to this article as no new data were created or analyzed in this study.
